# The Role of IL-17 and Related Cytokines in Inflammatory Autoimmune Diseases

**DOI:** 10.1155/2017/3908061

**Published:** 2017-02-20

**Authors:** Taku Kuwabara, Fumio Ishikawa, Motonari Kondo, Terutaka Kakiuchi

**Affiliations:** Department of Molecular Immunology, Toho University School of Medicine, Tokyo 143-8540, Japan

## Abstract

Interleukin-17 (IL-17) induces the production of granulocyte colony-stimulating factor (G-CSF) and chemokines such as CXCL1 and CXCL2 and is a cytokine that acts as an inflammation mediator. During infection, IL-17 is needed to eliminate extracellular bacteria and fungi, by inducing antimicrobial peptides such as defensin. This cytokine also plays an important role in chronic inflammation that occurs during the pathogenesis of autoimmune diseases and allergies such as human rheumatoid arthritis (RA) for which a mouse model of collagen-induced arthritis (CIA) is available. In autoimmune diseases such as RA and multiple sclerosis (MS), IL-17 is produced by helper T (Th) cells that are stimulated by IL-1*β* and IL-6 derived from phagocytes such as macrophages and from tissue cells. IL-17 contributes to various lesions that are produced by Th17 cells, one subset of helper T cells, and by *γδ* T cells and innate lymphoid cells. It strongly contributes to autoimmune diseases that are accompanied by chronic inflammation. Thus, a functional understanding of Th17 cells is extremely important. In this review, we highlight the roles of cytokines that promote the development and maintenance of pathogenic Th17 cells in autoimmune diseases.

## 1. Introduction

The immune system is a defense mechanism in the body that involves various types of blood cells derived from the bone marrow such as T cells and B cells, macrophages, and dendritic cells (DCs). The function of the immune system is to eliminate infectious microorganisms that have invaded the body and cancer cells that have been produced by mutations. The immune reaction leads to cell death under certain circumstances. Thus, excessive elimination of targets in chronic inflammatory reactions is harmful and is the cause of autoimmune diseases. Therefore, strict regulation is crucial to maintain immunological homeostasis. CD4-positive T cells, one type of T cell, are called helper T cells because they regulate the function of other immune cells. These helper T cells play a central role in the elimination of foreign microorganisms and in self-tolerance. Helper T cells produce cytokines that help activate immune cells in the microenvironment. IL-17 is an important cytokine not only for protective immunity against extracellular pathogens [1, 2], but also for the clearance of intracellular pathogens [3, 4]. In addition to its important role in protective immunity, IL-17 plays a critical role in the pathogenesis of various autoimmune inflammatory diseases. IL-17-producing cells, including *γδ*T cells, natural killer T cells, and innate lymphoid cells, are characterized by the expression of the transcription factor, retinoic acid receptor-related orphan receptor-*γ*t (ROR*γ*t) [5–7]. Dysregulation of protective immune responses causes autoimmune diseases. Self-reactive T cells are usually suppressed. When the balance of the self-reactive T cells and regulatory T cells is disturbed, the risk for autoimmune disease onset increases. High amount of production of IL-17 accompanied by excessive generation of Th17 cells may lead to autoimmune diseases. Although several types of cells produce IL-17, accumulated evidence has implicated an important role for Th17 cells in autoimmune diseases. This review summarizes the current knowledge on cytokines that control Th17 cell differentiation and cytokines that regulate Th17 cell functions by focusing on multiple sclerosis (MS), an autoimmune disease of the central nervous system (CNS), and the corresponding mouse model of experimental autoimmune encephalomyelitis (EAE).

## 2. IL-17-Producing Helper T Cells

### 2.1. Helper T Cells

**Figure 1:**
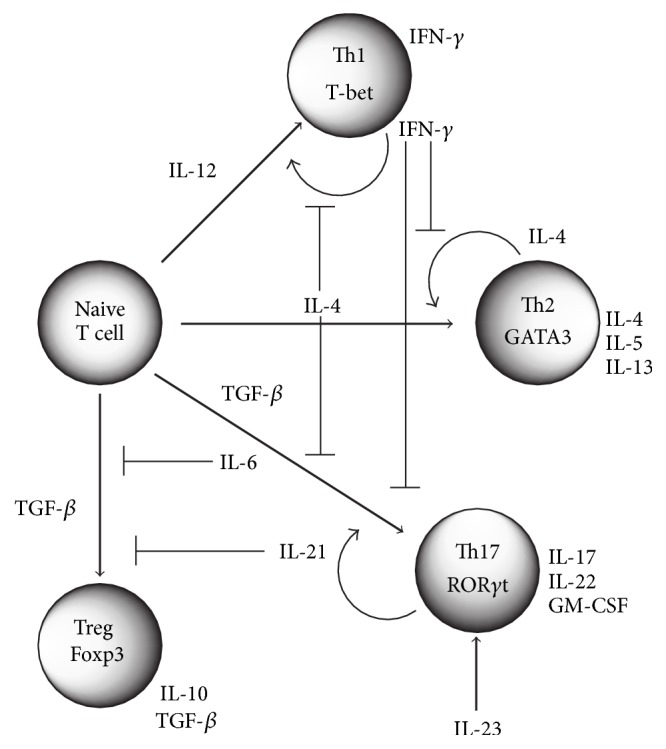
Regulation of Th cell differentiation. Naïve CD4 T cells differentiate into four distinct T cell subsets such as Th1, Th2, Th17, and induced T regulatory (Treg) cells dependent on the cytokine milieu.

Various hematopoietic and lymphoid progenitors are mobilized from the bone marrow and initiate T cell development in the thymus. During this process, they express an antigen receptor (the T cell receptor, TCR), and most cells differentiate into CD4-positive T cells or CD8-positive T cells. After completion of the maturation process, CD8-positive T cells circulate throughout the body, acquiring cytotoxic functions. They contribute to immunological homeostasis by killing cells that have been infected by viruses as well as cancer cells. On the other hand, CD4-positive T cells are helper T cells. They exhibit an immunological regulatory function. Helper T cells have previously been divided into mainly two subsets (Figure 1) [8]. Th1 cells differentiate under the influence of IL-12 and mainly produce interferon-*γ* (IFN-*γ*). IFN-*γ* strongly activates macrophages, promoting the elimination of intracellular pathogens. In other words, it supports cellular immunity in the acquired immune system. On the other hand, Th2 cells differentiate under the influence of IL-4. Th2 cells support B cells through IL-4 production. As a result, the antibodies produced by B cells switch their class from IgM to IgG or IgE. By inducing the production of IgG or IgE, elimination of extracellular parasites (such as nematodes) is promoted. While cellular immunity is performed by Th1 cells, Th2 cells support humoral immunity. Thus, although derived from the same precursor cells, when activated by antigen stimuli, helper T cells differentiate into subsets with different properties due to the surrounding environmental factors (in particular, cytokines). The mechanism of differentiation has been previously analyzed in detail to clarify their mutually exclusive properties. Namely, IFN-*γ* suppresses the differentiation of Th2 cells, while IL-4 inhibits Th1 differentiation. Therefore, the functional imbalance between Th1 and Th2 is at the origin of various immunological diseases. For example, when there is a bias toward Th1, autoimmune diseases such as MS and RA are more likely to occur, while if Th2 is dominant then allergic reactions represented by pollinosis are provoked. However, energetic research in recent years identified subsets other than Th1 and Th2 cells [9]. Among these, the Th17 cell subset, which produces IL-17, contributes to autoimmune diseases accompanied by chronic immune and inflammatory reactions, working together with Th1 cells.

### 2.2. Interleukin-17

IL-17 is a cytokine whose gene was isolated from a rat-mouse T cell hybridoma in 1993. Since it displayed a high degree of homology with the HVS13 Herpes virus gene, it was thought to be a subtype of the cytotoxic T-lymphocyte-associated protein (CTLA) family of proteins and called CTLA-8 [10]. In 1995, it was recognized as a new cytokine and named IL-17, and, today, six homologous molecules are known (IL-17A through IL-17F). To date, most studies focused on IL-17A, IL-17E, and IL-17F. IL-17A and IL-17F are highly homologous and share receptors. Thus, they have extremely similar functions. IL-17E is also known as IL-25, and because it presents low homology with other molecules of the family and contributes to the induction of allergies, it is thought to have functions different from those of IL-17A [11]. This review focuses on IL-17A. Several types of immune cells produce IL-17A, and Th17 cells, the newly established subset of helper T cells, have received particular attention [12–14]. Through the analysis of the function of Th17 cells in autoimmune diseases, there has been significant progress in the understanding of the biological significance of IL-17.

### 2.3. IL-17 Receptors and Their Signaling

IL-17 receptor A (IL-17RA) was identified as a new cytokine receptor for IL-17A and was found to be a member of the cytokine receptor family [15]. The IL-17 receptor family now consists of five members (IL-17RA, IL-17RB, IL-17RC, IL-17RD, and IL-17RE), all of which share sequence homology. All these receptors contain a fibronectin III-like domain in their extracellular region and a SEF/IL-17R (SEFIR) domain in their intracellular region [16]. IL-17RA had been long considered as the receptor of IL-17. However, newly emerged evidence suggests that IL-17RA is likely a common receptor used by the IL-17 family cytokines. Other receptors including IL-17RB, IL-17RC, and IL-17RE have been identified as specific receptors for IL-17E, IL-17A, and IL-17F and IL-17C, respectively. IL-17RD, originally identified as a negative regulator in fibroblast growth factor signaling, has recently been found to regulate IL-17A signaling [17, 18]. Recent studies have shown that IL-17A and IL-17F, believed to be predominantly expressed in Th17 cells, signal through a heteromeric receptor complex. This complex consists of IL-17RA and IL-17RC, which are single transmembrane proteins and ubiquitously expressed in various cell types, including epithelial cells, fibroblasts, and astrocytes [19–22]. It has been well documented that IL-17A and IL-17F can induce proinflammatory gene expression both alone and in synergy with TNF*α*, IL-6, G-CSF, IL-1, CXCL1, CCL20, and matrix metalloproteases [23–25]. IL-17 upregulates these proinflammatory genes through the activation of NF-*κ*B, MAPK, and C/EBP cascades [26]. Other studies demonstrated that IL-17 also activates the Jak-Stat and Jak-PI3 K pathways [27, 28]. In addition to altering gene expression, IL-17 stimulation can stabilize mRNAs. Normally, mRNA splicing factor (SF) is bound to mRNA to mediate its degradation. Upon IL-17 stimulation, mRNA is dissociated and stabilized [29, 30].

Adaptor protein Act1 and tumor necrosis factor receptor-associated factor (TRAF) regulate these molecular mechanisms after activation of IL-17R. Act1 was originally discovered as an NF-*κ*B activator [31, 32]. Several studies demonstrated that Act1 is recruited to the IL-17 receptor complex through the homotypic interactions of the SEFIR domains upon IL-17 stimulation [33, 34]. Act1 deficiency results in loss of IL-17-dependent NF-*κ*B activation and proinflammatory cytokine production. Following Act1 binding to the receptor complex, TRAF6 is recruited through interaction with the Act1 TRAF binding motif [35]. TRAF6 further activates downstream TRAF6-dependent TAK1 for NF-*κ*B activation.

IL-17-mediated mRNA stability of CXCL1 requires Act1 but not TRAF6, suggesting that Act1 mediates both TRAF6-dependent and TRAF6-independent pathways in IL-17R signaling [29]. In the TRAF6-independent pathway, TRAF2 and TRAF5 are required for IL-17-induced mRNA stabilization of CXCL1 [30]. Act1-TRAF5-TRAF2-SF complex formation induced by IL-17 prevents SF binding to CXCL1 mRNA for degradation. Phosphorylation of Act1 at Ser-311 was shown to be required for its association with TRAF2-TRAF5 complex. By mass spectrometry analysis, Act1 was found to be phosphorylated directly by the kinase IKK*ε* at Ser-311 upon exposure to IL-17. Consistently, IKK*ε* deficiency also prevented the formation of Act1-TRAF2-TRAF5 complex without disrupting interaction between TRAF6 and Act1, suggesting that phosphorylation of Act1 is necessary for the formation of the mRNA stabilization cascade. Thus, Act1 serves as a receptor proximal anchor platform for the initiation of two independent pathways activated by IL-17: (1) the TRAF6-dependent cascade for signaling and (2) TRAF2-TRAF5-dependent cascade for stabilization of mRNA.

Regulation of IL-17R pathways is important in the control of IL-17-mediated inflammation. As in intracellular mechanisms, fundamental features of inflammation have been revealed using mice lacking IL-17R subunits. IL-17RA-deficient fibroblasts failed to respond to IL-17A or IL-17F stimulation [36]. Subsequent studies showed that IL-17RC interacts with IL-17RA and it can bind to IL-17A or IL-17F. IL-17RC-deficient mice also failed to induce downstream gene expression in response to IL-17A and IL-17F and develop much milder disease than wild-type mice in experimental autoimmune encephalomyelitis [37–39]. These results suggest that the IL-17 receptor complex has a significant role in biological and pathological functions [39] and is a candidate therapeutic target. Therefore, identification of new compounds to block the activation of IL-17R is a powerful approach to prevent IL-17-mediated pathology. A monoclonal antibody targeting IL-17R would be a potential treatment to investigate this therapeutic tool for autoimmune diseases. Brodalumab, previously known as AMG827, is a human monoclonal antibody that binds to the human IL-17RA and blocks the biological activities of IL-17A and IL-17F. This antibody is a broad spectrum inhibitor of IL-17-mediated signaling pathways compared to other IL-17-targeted therapy. Brodalumab is currently being investigated in different phases of clinical trials for psoriasis (phase II) and for RA (phase I/II) [40, 41]. In a phase II study, brodalumab showed significant improvement in severe psoriasis compared to placebo [40]. In these preclinical studies, IL-17R targeted therapy did not show any major safety concern. However, further trials are warranted to confirm the safety profile of this therapy. IL-17 expresses protective properties against extracellular pathogens [42, 43]. Future trials with a large number of patients will provide more insight into the safety profile. Other therapeutic strategies against IL-17 are discussed below.

## 3. IL-17 and Autoimmune Diseases

Central immune tolerance is the thymic mechanism that eliminates self-reactive T cell receptor-producing cells. However, the elimination is not perfect. Thus, self-reactive T cells exist in peripheral tissues. Peripheral immune tolerance refers to the suppression of self-reactive T cells by regulatory T cells. Failure of both types of tolerance may result in autoimmunity and autoimmune diseases. In autoimmune diseases, the helper T cells that support the acquired immunity system attack tissues in an antigen-specific manner. Tissue dysfunction accompanied by localized organ damage induced by self-reactive T cells leads to diseases. In human diseases, it is difficult to identify antigens and the causes of disease. Thus, methods using mouse models of these diseases, in which the disease can be induced by the identified antigens, are often used as a strategy for studying autoimmune diseases. For example, the CIA model is often used as a model for human RA, and the EAE mouse model is often used as a model for human MS. Currently, Th17 cells have been established as having a close relationship with chronic inflammatory autoimmune diseases. In this section, the first half introduces the current knowledge on inflammatory cytokines related to Th17 cells in autoimmune diseases based on results obtained using the EAE mouse model, and the second half briefly explains the role of Th17 cells and IL-17 in RA and psoriasis.

### 3.1. Th17 Cells in EAE

MS is an autoimmune disease accompanied by chronic neuroinflammation that causes demyelination in the CNS. Accumulation of helper T cells is observed in the cerebrospinal fluid of patients. The EAE mouse model that recapitulates MS is an experimental system in which pathogenic helper T cells can be induced by administering antigens in the CNS tissue, resulting in limb paralysis. The pathophysiological analysis of EAE is useful for understanding MS. MS-related genes have been proposed by establishing and screening the disease-related genome analysis (genome-wide association studies, GWAS) database [44]. The results suggest that specific major histocompatibility complex (MHC) class II genes are associated with a significantly high risk for the onset of MS. Moreover, although somewhat less important than the MHC, a group of genes related to differentiation and activation of helper T cells also showed a significant relationship with MS. Thus, it is believed that this disease of the CNS is a T cell-mediated disease.

#### 3.1.1. Pathogenic T Cells: A Paradigm Shift from Th1 to Th17

Since Th1 cells strengthen cellular immunity and are present in chronic CNS inflammation [45], they and IFN-*γ* produced by them were thought to be important pathogenic factors in MS and EAE. IL-12 is a cytokine composed of p40 and p35 [46]. Since mice lacking p40 show resistance to EAE [47], the idea that Th1 cells are necessary for self-pathology in neuroinflammation has been supported. However, it is also known that mice lacking p35 and mice lacking the *β* chain of the IL-12 receptor as well as mice lacking IFN-*γ* and mice lacking the IFN-*γ* receptor show strong symptoms of EAE compared to wild-type mice [48–53]. Thus, the identification of helper T cells as the causative factor remained unclear. IL-23 is a cytokine composed of p19 and IL-12p40. Studies using mice lacking p19 shed light on the role of helper T cells in EAE [13, 54]. Incomplete induction of IL-17 producing helper T cells (Th17) was observed in mice lacking p19, without inhibition of Th1 cell differentiation. This mutation failed to induce EAE in mice, due to the lack of Th17 cells. EAE resistance inp40-deficient mice suggested that IL-23, but not IL-12, was necessary for the induction of neuropathogenic T cells. As a result, it is now believed that helper T cells that produce IL-17 are the cause of inflammatory autoimmune diseases such as EAE and MS.

#### 3.1.2. Cytokines Promoting Th17 Cell Development

After the discovery of the IL-23-Th17 axis, the properties of helper T cells were analyzed in detail. Th17 cells express RAR related orphan receptor *γ*t (ROR *γ*t) (encoded by Rorc), a master transcription factor [55]. IL-17, IL-22, and granulocyte-macrophage colony-stimulating factor (GM-CSF), effector cytokines produced by these cells, strongly contribute to tissue inflammation [13, 56–58]. The Th17 differentiation process was also gradually clarified and divided into two stages, a priming stage and a maturation stage.

Mice lacking p19 display resistance to autoimmune diseases and a reduction in Th17 cells, so the importance of IL-23 as a differentiation factor is established. However, IL-23 cannot induce naïve T cells into mature Th17 cells. Additionally, naïve T cells do not express IL-23R. These results suggested that the priming stage of Th17 cell differentiation was regulated by other factors. Multiple groups showed that, in mice, the induction of Th17 cells is possible through simultaneous stimulation with transforming growth factor (TGF) *β* and IL-6 [59–61]. In the presence of IL-6 at the priming stage, a naïve T cell is stimulated by cognate antigen to differentiate into an effector cell; then ROR *γ*t, IL-17, and IL-23R are expressed, and the direction toward a Th17 cell is decided. IL-6 also induces the expression of IL-1R. IL-1 *β*, an inflammatory cytokine, promotes the expression of ROR *γ*t through IRF4 pathway [62]. Therefore, mice lacking IL-1R cannot sufficiently induce Th17 cells and do not contract EAE [63]. Moreover, IL-1 *β* stimulation activates mammalian target of rapamycin (mTOR), promoting clonal expansion in an inflammatory environment [64]. TGF *β*, similarly to IL-6, is also a necessary factor for the early differentiation of Th17 cells, and this cytokine induces the expression of the master transcription factor, Foxp3, that is needed for the differentiation of regulatory T cells (Treg). Thus, it was unclear whether TGF *β* contributed to the differentiation into Th17 cells, since Th17 and Treg cells have different properties. Since IL-6 cannot induce Th17 cells on its own, it must occur together with TGF *β*. The contribution of TGF *β* was investigated. It was found that TGF *β*, working together with IL-6, induced the expression of both Foxp3 and ROR*γ*t [65]. The coexisting IL-6 suppresses Foxp3 expression by activating STAT3, thereby blocking Treg cell differentiation. However, based on recent research, it has been shown that it is possible to sufficiently induce Th17 cells with IL-6, IL-1*β*, and IL-23, and it became clear that TGF*β* is not required [66]. Th17 cells that have been induced by TGF*β* and IL-6 gradually decrease their IL-17 production until there are only unstable Th17 cells [67, 68]. It was shown by three independent research groups that IL-21 is necessary for continuous IL-17 production [67–69]. IL-21 is produced by Th17 cells while they are undergoing IL-6 stimulation-dependent differentiation, inducing the production of IL-17 and the expression of IL-23 receptors by autocrine action. IL-21 also contributes to the suppression of Foxp3 expression that is induced by TGF *β*, thereby promoting the differentiation of Th17 cells. This suppression of Foxp3 expression is also observed in mice lacking IL-6. Thus, IL-21 and IL-6 regulate Foxp3 expression by independent mechanisms. Recent studies reported that Th17 cells induced mainly by IL-6 and TGF *β* are effective at sites of extracellular parasitic bacteria and mucosa immunity but do not appear to cause autoimmune diseases accompanied by chronic inflammation. This shows that autoimmune diseases present Th17 cells that underwent a maturation process in order to acquire high pathogenicity.

Helper T cells that induce major tissue damage are closely related to severe autoimmune diseases. Thus, the types of factors necessary for the maturation stages after the initial priming processes were investigated. IL-23, said to be necessary for Th17 cell induction, and its contribution to the production of inflammatory cytokines that are found in autoimmune diseases such as EAE (MS) and CIA (RA) garnered the attention again. Studies using mice lacking IL-23 indicated that helper T cells, whose IL-23 signals are blocked, undergo an early differentiation process of Th17 cells producing ROR*γ*t and IL-17 [70]. This process depends on TGF*β* and IL-6, not on IL-23. On the other hand, Th17 cells induced in a manner independent of IL-23 are not capable of sufficient clonal expansion, thus the pathogenicity of Th17 cells is weak, resulting in mild to no tissue damage. Self-reactive helper T cells are suppressed by immunosuppressive cells such as Treg cells. Recent studies clarified that, after IL-23 stimulation, Th17 cells avoid immunosuppressive cells [71, 72]. IL-27 is a suppression mediator. Stimulation by IL-27 activates the transcription factor STAT1. STAT1 activation inhibits the activity of STAT3, which is needed for Th17 cell differentiation [71]. IL-23 stimulates Th17 cells to suppress the expression of the IL-27 receptor [71, 73]. Therefore, IL-23-mediated Th17 cells are resistant to IL-27. GWAS results also show that IL-23 is genetically linked to chronic inflammatory autoimmune diseases [74]. The results of this genome analysis support the importance of IL-23.

As described above, from many analyses, TGF*β* and IL-6 are necessary factors for the early stage of Th17 cell differentiation, while IL-23 plays a central role in the functional maturation and maintenance of autopathologic Th17 cells.

#### 3.1.3. Mechanisms Underlying the Infiltration of Th17 Cells into the CNS

Blood cells in the CNS, including cells responsible for immunity as well as many blood proteins cannot pass through the blood-brain barrier (BBB) that strictly limits the flow of substances like proteins and cells from the bloodstream into the CNS. Cell migration and the transfer of the necessary proteins (nutrients) from the blood into the CNS are performed in an active way. This machinery requires energy. The structure of the BBB includes vascular endothelial cells that are strongly joined by tight junctions and various types of underlying cells such as pericytes, astrocytes, and microglia [75, 76]. The role of the BBB is to separate the CNS from the permanent changes in peripheral tissues caused by infection by external microorganisms, thereby maintaining a safe microenvironment for neurons. Pathogenic T cells must cross the BBB by some mechanisms that weaken the BBB's defenses. In such a disease, for autoreactive helper T cells to infiltrate the CNS, they destroy the tight junctions of the BBB in an IL-17-dependent manner [77]. Studies using whole-mount-section analysis of EAE mice showed that self-reactive helper T cells locally accumulate behind the fifth lumbar vertebra (L5) [78]. An inflammatory cycle due to IL-17, produced by Th17 cells, and IL-6, produced by vascular endothelial cells, is formed locally at the L5 vertebra, and the vascular endothelial cells respond to this in a cyclic way to express the chemokine CCL20. As a result, Th17 cells that express the chemokine receptor CCR6 locally accumulate and pass through the tight junctions that are damaged by the inflammatory cycle [79]. Through such processes, encephalitogenic Th17 cells pass through the BBB and arrive at the CNS, which is the target. After onset, at L5, various chemokines other than CCL20 are produced. Thus, once the inflammatory cycle has occurred, many more cells will infiltrate.

#### 3.1.4. The CCR7 Ligand Is Necessary for the Induction of Pathogenic Th17 Cells

The chemokines that are produced at L5, the entry site of immune cells, include CCL21. Alt et al. presented a model in which pathogenic T cells infiltrate the CNS in a manner dependent on CCR7, the receptor for CCL21. They reported that the CCL21-CCR7 signal is necessary for EAE [80]. Since EAE does not occur in mice lacking CCL21 or in CCR7 knockout mice, this model was initially considered valid [81]. However, when pathogenic Th17 cells are adoptively transferred into mice lacking CCL21 or into wild-type mice, both groups show similar symptoms and rates of onset. Thus, there is no direct relationship between CNS migration of TH17 cells and CCL21. It is thought that the CCR7 ligand has an indispensable role in the disease at a step other than the infiltration into the CNS. When CCR7 knockout mice and mice lacking CCL21 were studied in detail, it was found that CCL21 stimulates DCs to strongly induce IL-23 production [81]. It was clarified that, in mice lacking CCL21, there is a reduction in IL-23 production and blocking of the induction of Th17 cells. Since the differentiation into Th2 cells was similar to that of wild-type mice, it is thought that CCL21-CCR7 stimulation is specific to a Th subset. DCs migrate into lymph nodes in a manner dependent on CCL21. This migration is dependent on ERK. However, the production of IL-23 is dependent on the Akt pathway [82]. CCR7 ligands direct DC functions between cellular migration and IL-23 production. These DC functions are switched by this chemokine and microenvironment.

### 3.2. Th17 in RA and Psoriasis

As for MS and EAE, Th17 cells strongly contribute to RA. Since Th17 cells cannot be separated from RA and MS disease conditions, the role and function of IL-17 have been analyzed in detail. The important clinical issue for RA is bone and joint destruction. Since bone destruction is directly related to changes in the joint structure, treatment for prevention of joint destruction is very important. Osteoclasts are the only cells that break down the bone, and they are a specially differentiated type of macrophage. Osteoclasts differentiate from monocytes, and receptor activator of nuclear factor kappa-B ligand (RANKL) promotes this process [83, 84]. Studies focused on how helper T cells that infiltrate the joints induce osteoclastogenesis from monocytes. Helper T cells that produce IL-17 have been found in the synovial tissue of patients with RA [85]. IL-17 stimulates the osteoblasts in the joints, inducing RANKL expression. Then, through the interaction with osteoblasts, monocytes respond to RANKL and mature into osteoclasts. After this mechanism was reported, interest in IL-17 for RA and its animal models grew [86, 87]. The identity of the helper T cells that regulate IL-17 production in the synovium remained unclear, but, after the discovery of Th17 cells as a new subset in 2006, the RANKL-dependent induction mechanism of osteoclasts via Th17 cells in RA became clear [88]. In RA and CIA, continuing inflammation is an important disease condition, similar to bone destruction. IL-22 produced by Th17 cells certainly regulates chronic inflammation. IL-22 stimulates synovial fibroblasts to induce cell proliferation and the production of inflammatory chemokines [89]. Studies using a mouse model also showed that IL-22 induces osteoclastogenesis [90]. Moreover, IL-6 produced by fibroblasts responding to IL-17 derived from Th17 cells amplifies inflammation [79]. IL-6 stimulates synovial tissue in an autocrine manner, worsening the condition by amplification cycle that releases inflammatory mediators. Thus, IL-17 maintains the inflammatory cycle via downstream cytokines. It has recently been reported that IL-17 in the synovial tissue is not derived from Th17 cells, but rather from mast cells [91]. That does not change the importance of IL-17 for the disease condition, but the possibility remains that the role of Th17 cells in this disease will be revised.

Psoriasis is a chronic inflammatory skin disease characterized by hyperproliferation and abnormal differentiation of epidermal cells. Pronounced acanthosis and inflammatory infiltration such as of neutrophils and lymphocytes are observed in lesions. As cyclosporin, a calcineurin inhibitor, shows therapeutic effectiveness, it is thought that T cells contribute to psoriasis pathology. Recent studies clarified that Th17 cells contribute to the onset of psoriasis [92, 93]. Th17 cells that have infiltrated the skin produce not just IL-17, but also IL-22. IL-22 stimulates keratocytes in the skin, leading to the activation of STAT3, followed by the proliferation of keratinocytes and acanthosis [94]. IL-17 and IL-22 induce keratinocytes to express CXCL1 and CXCL8, inducing further cell infiltration [94–98]. Thus, a positive psoriasis feedback loop is formed. A contribution of IL-36 to the detailed mechanism of this positive feedback loop has been suggested [99]. IL-36 is produced by keratinocytes and stimulates resident DCs that are normally present in the skin. In response to IL-36, DCs express IL-23, which appears to increase the pathogenicity of Th17 cells [100]. In the normal skin, an IL-36R antagonist is expressed, to compete with IL-36 stimulation [101]. However, it is thought that, as the disease condition progresses, the suppression by the IL-36R antagonist is abrogated.

### 3.3. Plasticity of Pathogenic Helper T Cells

In chronic inflammation, IL-23 stimulation confers higher pathogenicity on Th17 cells, and, in this process, not just IL-17, but also IFN*γ*, IL-22, and GM-CSF are produced. In a recent study, based on the way functional cytokines are produced, helper T cells have been divided into multiple subsets such as Th9 cells and Th22 cells [2]. Th1 cells and Th2 cells stably maintain their properties. In contrast, it seems that Th17 cells infiltrated into CNS lesions produce various cytokines and differentiate from Th17 cells into other subsets [102]. Studies using an IL-17 reporter mouse indicated that, during EAE onset, cells that produced IL-17 were converted into cells that produced IFN*γ* [103]. Based on this result, a model was proposed in which Th17 cells redifferentiated either into Th17/Th1 hybrid cells, or into a type of Th1 cells that stopped producing IL-17 (ex-Th17). This model may explain the presence of Th1/Th17 hybrid cells in the CNS from patients with MS. Moreover, these cells producing IFN*γ*, which may be ex-Th17 cells, are more pathogenic than Th17 cells. IFN*γ* can be produced by conventional Th1 cells and ex-Th17 cells. However, conventional Th1 cells are not exposed to IL-23 and, therefore, do not express IL-1R, while ex-Th17 cells exposed to IL-23 in the past can express IL-1R. Therefore, the expression of IL-1R helps distinguishing between IFN*γ*-producing ex-Th17 cells and conventional Th1 cells. Since GM-CSF is encephalitogenic, a new subset of cells has been named ThGM-CSF [56, 57]. According to this model, it is highly possible that GM-CSF-producing helper T cells acquire the ability to produce GM-CSF after Th17 cells stop producing IL-17. In fact, helper T cells that produce both IL-17 and GM-CSF have been observed [104]. Additionally, there are cases where other subsets turn into Th17 cells. TGF*β*, which is necessary for the differentiation of Th17 cells, is a cytokine that induces the differentiation of Treg cells. Komatsu et al. using a CIA mouse model showed that Foxp3-positive Treg cells in the joint synovia redifferentiated into Th17 cells (IL-17-producing ex-Treg cells) [105]. IL-17-producing ex-Treg cells express RANKL and cause strong osteoclast differentiation compared to the normal type of Th17 cells. These monocytes interact directly with IL-17-producing ex-Treg cells, not via synovial fibroblasts.

## 4. Autoimmune Disease Treatment Targeting Th17 Cells

**Figure 2:**
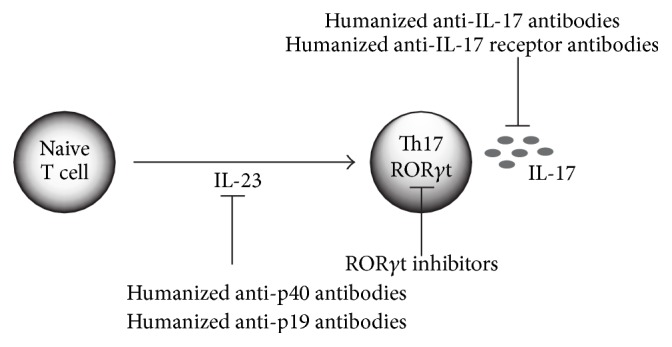
Tools for targeting the Il-23-Th17 axis. Various therapeutic tools are available to target the interlukin-23-Th17 pathway. Ustekinumab and briakinumab are two monoclonal antibodies that target p40, and tildrakizumab and guselkumab are monoclonal antibodies that target p19. Inhibitors of Th17 cell generation target RORgt. Ixekizumab and secukinumab are monoclonal antibodies that target IL-17, and brodalumab is a monoclonal antibody that targets IL-17 receptor A.

Based on research results using GWAS and animal models, clinical trials have begun, targeting either IL-23, which contributes to the final differentiation and function acquisition of pathogenic Th17 cells, or ROR*γ*t, which is a master transcription factor for Th17 cells, or IL-17, which is an effector cytokine [74, 106] (Figure 2). With regard to autoimmune diseases such as RA, ankylosing spondylitis, chronic inflammatory intestinal diseases, psoriasis, and MS, clinical research is progressing on humanized anti-IL-23 antibodies, humanized anti-IL-17 antibodies, and humanized IL-17R antibodies, and some extremely hopeful results have been obtained. Among these, a high improvement rate has been observed in the clinical symptoms of psoriasis compared to existing treatment methods [107–110].

Screening tests are being performed to identify small molecules regulating the functions of Th17 cells. Among molecules that contribute to the maturation and function of Th17 cells, ROR*γ*t has DNA binding sites and ligand binding regions, and therefore it is thought to be easy to focus on the targeted sites. ROR*γ*t is also different from other transcription factors in that it shows hemocyte-specific expression. Thus, side effects might be minimized in biological applications. The cardiac glycoside, digoxin, which has been used to treat heart disease, has been screened as a ROR*γ*t-specific inhibitor [111]. Digoxin inhibits the differentiation of Th17 cells without inhibiting the differentiation into other subsets. Digoxin derivatives that are derived from this cardiac glycoside specifically inhibit the production of IL-17 in human and mouse Th17 cells. The small molecule, SR100, which is a ligand of the liver X receptor LXR, one of the endonuclear receptors expressed in the liver, specifically binds to the ligand binding domain of ROR*γ*t [112]. As a result, there is a change in ROR*γ*t structure suppressing its interaction with a coactivator that increases its transcription activity, thereby inhibiting Th17 cell differentiation. Ursolic acid, a natural compound, selectively binds to ROR*γ*t, suppressing not only the Th17 cell differentiation process, but also the functions of Th17 cells after differentiation. Administration to EAE mice reduced limb paralysis [113]. Although these results were obtained in an animal model, ursolic acid shows promise, and one expects such development for all compounds that similarly bind specifically to ROR*γ*t.

## 5. Conclusions

In this review, we discussed IL-17 and related cytokines in chronic autoimmune diseases. Since the discovery of IL-17, there have been many reports that Th17 cells are important in human and mouse chronic autoimmune diseases. While Th17 cells and IL-17 directly lead to the worsening of RA disease conditions, they are important factors that can be targeted to alleviate diseases. There is hope that molecular therapy targeting IL-23 or the master transcription factor ROR*γ*t, which are necessary for autoimmune pathology, although they are unnecessary for the differentiation process of Th17 cells, will improve symptoms at the biological level. It is also predicted that multiple approaches will expand the range of application to other diseases in the future. If the molecular therapy strategies that have succeeded with helper T cells that produce IL-17 can also be applied to other helper T cells then they may be applied to not only diseases that are due to excessive immune response such as allergies, but also to immunodeficiency recovery and cancer immunity. In this review, we provided an overview of the role of IL-17 and Th17 cells in autoimmune diseases and hope that we have stimulated the reader's scientific sensitivity.

## Abbreviation

BBB: Blood-brain barrier
CD: Cluster of differentiation
CIA: Collagen-induced arthritis
CNS: Central nervous system
CTLA: Cytotoxic T-lymphocyte-associated protein
EAE: Experimental autoimmune encephalomyelitis
G-CSF: Granulocyte colony-stimulating factor
GM-CSF: Granulocyte-macrophage colony-stimulating factor
GWAS: Genome-wide association studies
IFN: Interferon
Ig: Immunoglobulin
IL: Interleukin
MHC: Major histocompatibility complex
MS: Multiple sclerosis
mTOR: Mammalian target of rapamycin (mechanistic target of rapamycin)
RA: Rheumatoid arthritis
RANKL: Receptor activator of nuclear factor kappa-B ligand
ROR*γ*t: RAR related orphan receptor *γ*t
STAT: Signal transducers and activators of transcription
TGF: Transforming growth factor
Th: Helper T.
